# Successful repair of left ventricular rupture with pseudoaneurysm: a case report

**DOI:** 10.1093/jscr/rjad444

**Published:** 2023-08-08

**Authors:** Abraham G Tiruneh, Admikew Bekele, Yidnekachew Asrat, Qaleab Tsegaye, Workneh Tesfaye, Abebe Bezabih

**Affiliations:** College of Health Sciences, Addis Ababa University, Addis Ababa, Ethiopia; College of Health Sciences, Addis Ababa University, Addis Ababa, Ethiopia; College of Health Sciences, Addis Ababa University, Addis Ababa, Ethiopia; College of Health Sciences, Addis Ababa University, Addis Ababa, Ethiopia; College of Health Sciences, Addis Ababa University, Addis Ababa, Ethiopia; College of Health Sciences, Addis Ababa University, Addis Ababa, Ethiopia

**Keywords:** Ventricular rupture, Pseudoaneurysm, Dacron patch repair

## Abstract

Ventricular rupture with pseudoaneurysm is a rare phenomenon that usually occurs after myocardial infarction, previous cardiac surgery and infectious or inflammatory conditions. To prevent rupture of the pseudoaneurysm, urgent repair is recommended. We report successful open surgical repair of a 46-year-old man, who presented with pseudoaneurysm communicating with left ventricle.

## INTRODUCTION

Left ventricular (LV) pseudoaneurysm is a rare condition that forms after ruptured ventricle is contained by pericardium or scar tissue [1]. While free intrapericardial rupture leads to cardiac tamponade and death, rarely the cardiac rupture can be contained and form pseudoaneurysm avoiding immediate tamponade [1]. In this paper, we report a successful surgical repair of LV rupture that formed pseudoaneurysm.

## CASE REPORT

**Figure 1:**
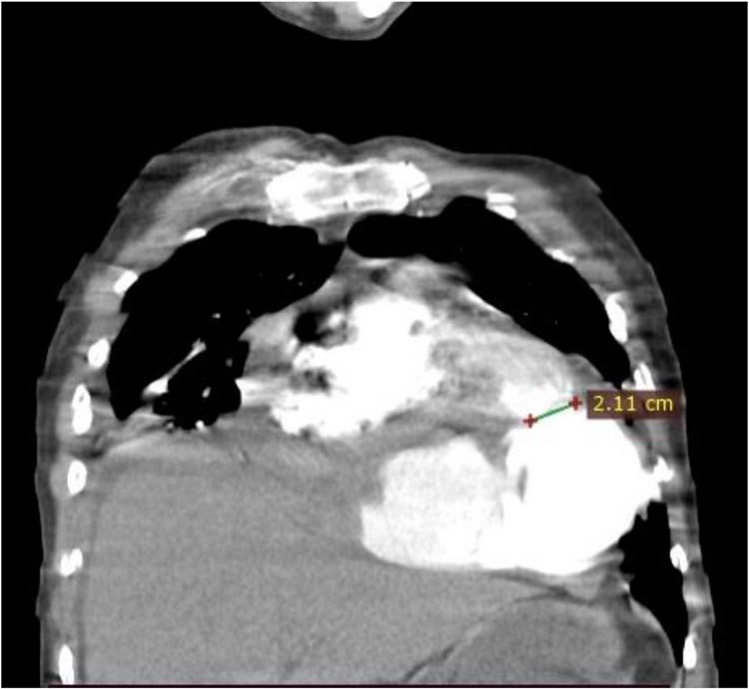
Coronal CT scan image. The figure shows a 2.1 cm communication between left ventricle and pseudoaneurysm. The heart is pushed upwards.

**Figure 2:**
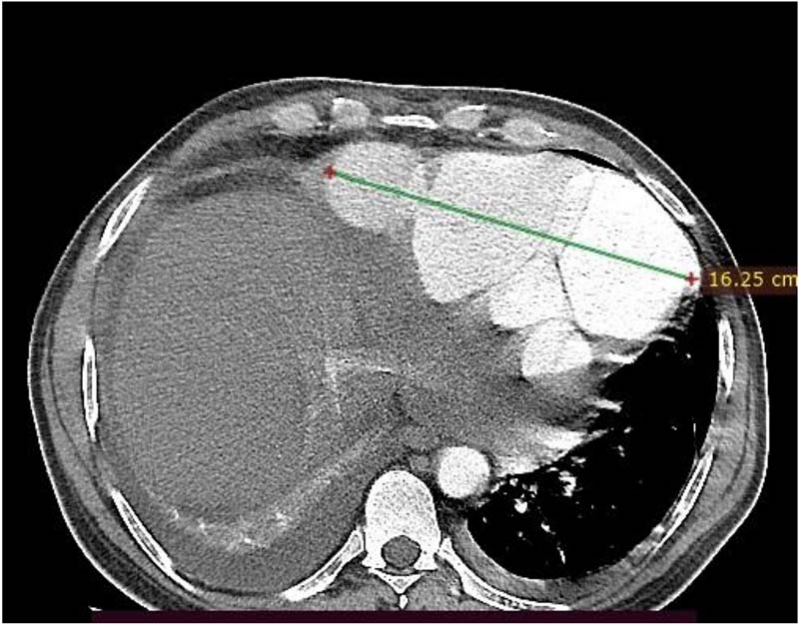
Axial image, just below the lower level of the heart, shows a 16 cm septated pseudoaneurysm that enhances with contrast.

A 46-year-old man from rural Ethiopia was referred 7 days after he started to have sudden shortness of breath, chest pain and cough. On 6th day of his symptoms, he had brief loss of consciousness and left-sided body weakness. When he arrived to our hospital on 7th day of symptom onset, his vitals were: blood pressure 100/70 mmHg, pulse rate 104–112 beat/min, respiratory rate 24 breaths/min, SpO2 95% on room air. There was Grade 3 systolic murmur at the apex. Power of left upper and lower extremities was 3 and 4 out of 5, respectively. Laboratory tests were white cell count of 15 800, with 89% neutrophils, hemoglobin of 11.4 g/dl, platelet count of 180 000, INR of 2.2. Cardiac markers and Electrocardiogram (ECG) were normal. Echocardiography showed 19 mm defect at apex of left ventricle with bidirectional flow between ventricular chamber and pseudoaneurysmal septated intrapericardial collection. Chest CT revealed huge pseudoaneurysm (contained rupture) the communicates with left ventricle via a 2.1 cm rupture (see Figs 1 and 2). Patient’s medical history was relevant for unprovoked deep venous thrombosis (DVT) of left lower extremity a year back, which was treated with warfarin.

**Figure 3:**
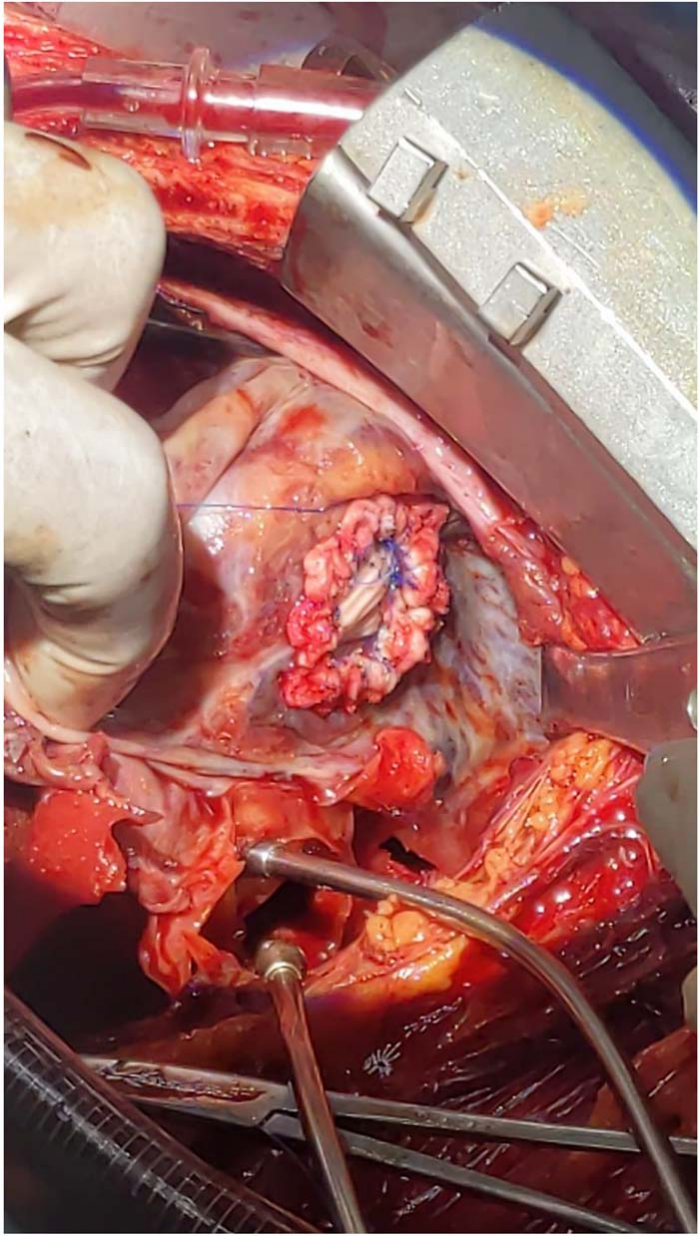
Intraoperative picture of a 2 cm rupture of left ventricle repaired with Dacron patch.

With diagnosis of embolic stroke and LV pseudoaneurysm, he was stabilized in cardiac intensive care unit, and then operated via median sternotomy. Pericardium appeared edematous and darkened because of underlying hematomata. To prevent uncontrolled bleeding, extrapericardial cannulation was done on ascending aorta. Then pericardium covering right atrium was then opened and superior vena cava was cannulated and bypass was initiated. When pericardium over the pseudoaneurysm was then opened, there was a huge cavity measuring about 16 cm transverse and 10 cm vertically, which had pushed the heart upward. There was some thrombus inside. It was freely communicating with inferior wall of LV close to apex through a 2 cm rupture. The cavity wall and coagulated hematoma were removed and perforation repaired with Dacron patch (see Fig. 3). Chest tube and mediastinal tube were left and sternum was closed. He had smooth postoperative course with normal postoperative echocardiography. He stayed for 12 days in the ICU and was discharged on 18th postoperative day. He did not require any cardiac medications.

## DISCUSSION

In the largest review of case reports of LV pseudoaneurysm that included 290 patients, the most common causes of LV pseudoaneurysm formation were myocardial infarction (55%), previous cardiac surgery (33%) and trauma (7%) [1]. In the setting of myocardial infarction, pseudoaneurysm occurs in <1% of patients, mostly in inferior or posterior location owing to possible reason that anterior rupture results in death from tamponade. Patients usually present within 2 months [1, 2]. Other rare causes like viral infection, tuberculosis and systemic lupus erythematosus have also been reported [3, 4]. Our case is peculiar in that it was not possible to identify the cause with certainty, which may be because of his late presentation at which time markers of myocardial infarction may be normal even after an event, however, sudden onset of chest pain, his previous unprovoked deep venous thrombosis, and the location of ventricular rupture suggests the most likely cause is myocardial infarction.

LV pseudoaneurysms mostly present with symptoms; however, about 10% may be asymptomatic [1]. Symptoms of heart failure, chest pain, dyspnea and cerebrovascular accident represent most common presentation [1, 5, 6]. Murmur is reported in up to 70%. About 40–65% of patients develop thrombus from the stasis, and embolism occurs in 5% [7]. In one case report, 72-year-old man with no history of coronary artery disease presented with left middle cerebral artery stroke and was later found to have LV pseudoaneurysm with thrombus [5]. Our patient had also a hemiparesis from embolic stroke that later improved.

Workup should include ECG and echocardiography. In setting MI-associated pseudoaneurysm, about 95% have abnormal ECG, mostly nonspecific ST changes [1]. Transthoracic echocardiography can show turbulent flow and communication between pseudoaneurysm and LV. MRI, CT scan and angiography are also important in differentiating true aneurysm from pseudoaneurysm, localizing and quantifying the pseudoaneurysm, as well as surgical planning. In our case, echocardiography clearly showed the communication. Cardiac MRI and angiography are not available; therefore, CT scan was done. CT was important to plan the surgery, to avoid entry into the cavity during sternal opening and before aortic cannulation. Angiography is important to map coronary arteries and do concomitant CABG with repair of rupture [2].

Even though true natural history of LV pseudoaneurysm is difficult to determine because of publication bias, surgery offers better outcome than medical treatment alone. Surgery is a recommended treatment of choice as LV pseudoaneurysms have high risk of rupture (30–45%) and mortality of about 48% with medical treatment alone [6, 8]. Surgical mortality has been decreasing, with latest report being <10% [1, 9]. Surgical approach should be planned beforehand with imaging. Securing aortic and venous cannulations to go on bypass is critical before opening the pseudoaneurysm. In our case, extrapericardial aortic cannulation was initially done to avoid opening the pericardium. The site of rupture can be repaired with pledgeted suture or graft (Dacron, Gor-Tex, pericardium) [5].

## CONCLUSION

LV pseudoaneurysm occurs when ruptured ventricle is contained with scar tissue or pericardium. It is usually a complication of myocardial infarction, however, it can occur in patients without evidence of myocardial infarction, especially if they present late when myocardial markers might be negative. It is prone to rupture, and the rupture site should be repaired under cardiopulmonary bypass with pledgeted suture or graft. Caution must be taken not to enter pseudoaneurysm cavity before cannulation, which otherwise could lead to uncontrolled bleeding.

## Data Availability

All data used for this report can be accessed at request to corresponding author.

## References

[1] Frances C, Romero A, Grady D. Left ventricular pseudoaneurysm. J Am Coll Cardiol 1998;32:557–61. 9741493 10.1016/s0735-1097(98)00290-3

[2] Varghese S, Ohlow MA. Left ventricular free wall rupture in myocardial infarction: a retrospective analysis from a single tertiary center. JRSM Cardiovasc Dis 2019;8:204800401989669. 10.1177/2048004019896692 PMC6923527 31903187

[3] Gumrai P, Na-Nan K, Tepsuwan T, Suwannasom P, Louthrenoo W. Cardiac wall rupture in systemic lupus erythematosus: a case report and review of the literature. Acta Rhumatol 2023;42:2223–9. 10.1007/s10067-023-06614-8 37140686

[4] Borrhomée S, Vergnat M, Roussin R, Hascoët S. A rare case of left ventricular pseudoaneurysm due to tuberculosis in a 13-year-old boy. World J Pediatr Congenit Heart Surg 2019;10:370–2. 29157124 10.1177/2150135117716422

[5] Incognito C, Parker J, Arustamyan M, Matta M, Posadas K, Lee R. Large left ventricular pseudoaneurysm presenting as an embolic stroke after a ‘silent’ myocardial infarction. Tex Heart Inst J 2023;50:e227922. 36988947 10.14503/THIJ-22-7922 PMC10178654

[6] Alapati L . Left ventricular pseudoaneurysm: a case report and review of the literature. World J Clin Cases 2014;2:90. 24749118 10.12998/wjcc.v2.i4.90 PMC3985042

[7] Reeder GS, Lengyel M, Tajik AJ, Seward JB, Smith HC, Danielson GK. Mural thrombus in left ventricular aneurysm: incidence, role of angiography, and relation between anticoagulation and embolization. Mayo Clin Proc 1981;56:77–81. 7464234

[8] Caldeira A, Albuquerque D, Coelho M, Côrte-Real H. Left ventricular pseudoaneurysm: imagiologic and intraoperative images. Circ Cardiovasc Imaging 2019;12:e009500. 31766861 10.1161/CIRCIMAGING.119.009500

[9] Brown SL, Gropler RJ, Harris KM. Distinguishing left ventricular aneurysm from pseudoaneurysm. Chest 1997;111:1403–9. 9149600 10.1378/chest.111.5.1403

